# Detection of *Brucella abortus* DNA in aborted goats and sheep in Egypt by real-time PCR

**DOI:** 10.1186/s13104-015-1173-1

**Published:** 2015-06-03

**Authors:** Gamal Wareth, Falk Melzer, Herbert Tomaso, Uwe Roesler, Heinrich Neubauer

**Affiliations:** Friedrich-Loeffler-Institut, Federal Research Institute for Animal Health, Institute of Bacterial Infections and Zoonoses, Naumburger Str. 96a, 07743 Jena, Germany; Institute of Animal Hygiene and Environmental Health, Free University of Berlin, Robert-von-Ostertag Str. 7-13, 14163 Berlin, Germany; Department of Pathology, Faculty of Veterinary Medicine, Benha University, Qalyobia, Egypt

**Keywords:** *Brucella abortus*, Cross-species transmission, Real-time PCR, Small ruminants

## Abstract

**Background:**

Brucellosis is a major zoonoses affects wide range of domesticated as well as wild animals. Despite the eradication program of brucellosis in Egypt, the disease is still endemic among cattle, buffaloes, sheep, goats, and camels.

**Results:**

In the present study, abortion occurred naturally among 25 animals (10 cows, 5 buffaloes, 9 Egyptian Baladi goats and 1 ewe) shared the same pasture were investigated by real-time polymerase chain reaction (RT-PCR). DNA of *Brucella* (*B.*) *abortus* was detected in serum of goats and sheep which has aborted recently by species-specific RT-PCR. The results suggest cross-species infection of *B. abortus* from cattle to non-preferred hosts raised in close contact.

**Conclusion:**

This article will renew our knowledge about the *Brucella* agent causing abortion in small ruminants in Egypt. Information provided in this study is important for surveillance program, because eradication programs and vaccination strategies may have to be adapted accordingly.

## Background

Brucellosis is a serious zoonosis transmitted by direct contact to secretions of animals which have aborted or contaminated dairy products [1]. The genus *Brucella* (*B*.) is a facultative intracellular pathogen that currently includes 11 accepted nomo-species. Based on the primary host species specificity. The ‘classical’ six species are *B. melitensis*, *B. abortus*, *B. suis*, *B. canis*, *B. ovis*, and *B. neotomae* which are primarily isolated from small ruminants, bovines, pigs, dogs, sheep and desert wood rats, respectively [2]. Two species of marine origin (*B. pinnipedialis* from seals, and *B. ceti* from dolphins and whales). *B. microti* was isolated from the common vole *Microtus arvalis* in middle Europe [3, 4]. *B. inopinata* was isolated from a breast implant wound of a North American female patient [5]. Recently, *B. papionis* was isolated from baboons (*Papio* spp.) [6].

In Egypt, brucellosis is still endemic and infects a wide range of animal species causing tremendous economic losses [7]. *B. melitensis* was isolated from cattle, buffalo, sheep, goat and Nile catfish in the past [8, 9]. In contrast, *B. abortus* was isolated from cattle, buffalo and camel [10–12], but was not recorded in small ruminant [13]. Host specificity of *Brucella* pathovars has been recognized for a long time and was used to phenotype isolates in the past. Goats and sheep are considered the classical and preferred hosts for *B. melitensis*. The clinical, pathological and epidemiological picture of caprine brucellosis due to *B. melitensis* is similar to *B. abortus* infection in cattle [1]. Due to existence of mixed livestock shelters and uncontrolled animal flock movements in Egypt [8], it was considered necessary to investigate the ability of *Brucella* isolates to be transmitted to and replicate outside its preferred host species in field conditions. Therefore, the present study was performed to investigate whether interspecies transmission of *B. abortus* may occur naturally and may cause clinical disease in small ruminants. This is of important once, because current eradication programs and vaccination strategies may have to be adapted if trans-species infections play a relevant role.

## Results

A storm of abortion occurred naturally among ten cows (*Bos taurus*), five buffaloes (*Bupalus bubalis*), nine Egyptian Baladi goats (*Capra hircus*) and one ewe (*Ovis orientalisaries*). Aborted animals submitted to veterinary clinic after abortion for diagnosis and treatment in a small village at Minufya governorate, Delta region, Egypt. All aborted animals shared the same pasture, but were owned by different peasants from neighboring localities. Serum samples were collected from animals after receiving permission from the owners. Samples from aborted fetus were not available. Sera were analyzed using the rose bengal test (RBT), the complement fixation test (CFT) and enzyme linked immunosorbent assay (ELISA) (IDEXX Brucellosis serum X2 AB test, Montpellier SAS, France).

Genomic DNA was extracted with the High Pure template preparation kit (DNA HP kit, Roche Applied Sciences, Mannheim, Germany) according to the manufacturer’s instructions. Specific real-time PCR assays for genus and species described by Probert et al. were performed in single runs [14]. The primers and probes were obtained from TIB MOLBIOL (Berlin, Germany) (Table 1). Each amplification reaction mixture was contained 0.75 μl of each primer (0.3 μM), 12.5 μl TaqMan™ Universal Master Mix (Applied Biosystems, USA), 0.25 μl probe (0.1 μM), 2 μl of DNA template and was filled up to a total volumes of 25 μl with HPLC grade water. Positive controls that contained *Brucella* DNA and no template controls (NTC) that contained PCR-grade water instead of DNA were used in all assays. Real-time-PCR assays were performed with the following cycling conditions, decontamination at 50°C for 2 min, one cycle with initial denaturation at 95°C for 10 min, and 50 cycles with 95°C for 25 s and 57°C for 1 min. All samples were tested in duplicates; cycle threshold (ct) values below 40 cycles were interpreted as positive.

**Table 1:** Primers and specific probes used in the real-time multiplex PCR assay for the detection of *Brucella* spp., *B. abortus*, and *B. melitensis*

PCR Identification	Primer and probe	
*Brucella* spp.	Forward primer 5′–3′	GCT-CGG-TTG-CCA-ATA-TCA-ATG-C
	Reverse primer 5′–3′	GGG-TAA-AGC-GTC-GCC-AGA-AG
	Probe 5′–3′	6FAM-AAA-TCT-TCC-ACC-TTG-CCC-TTG-CCA-TCA-BHQ1
*B. abortus*	Forward primer 5′–3′	GCG-GCT-TTT-CTA-TCA-CGG-TAT-TC
	Reverse primer 5′–3′	CAT-GCG-CTA-TGA-TCT-GGT-TAC-G
	Probe 5′–3′	HEX-CGC-TCA-TGC-TCG-CCA-GAC-TTC-AAT-G-BHQ1
*B. melitensis*	Forward primer 5′–3′	AAC-AAG-CGG-CAC-CCC-TAA-AA
	Reverse primer 5′–3′	CAT-GCG-CTA-TGA-TCT-GGT-TAC-G
	Probe 5′–3′	Cy5-CAG-GAG-TGT-TTC-GGC-TCA-GAA-TAA-TCC-ACA-BHQ2

**Figure 1:**
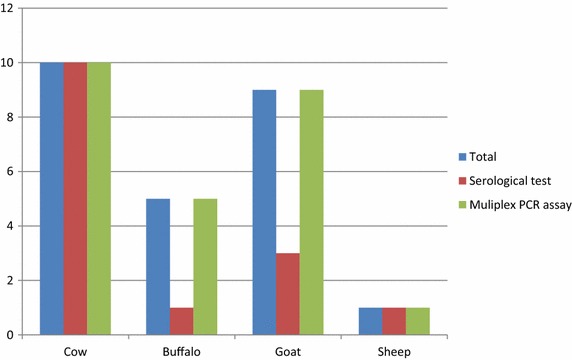
Serological and Multiplex PCR assay result in cow, buffalo, goat and sheep.

Serum samples collected very recently after abortion from four buffaloes and six goats gave negative results in serology. Contrastingly, samples collected 3 weeks after abortion produced strong positive reactions in RBT, CFT and ELISA. Real time-PCR assays resulted in a higher numbers of positive cases than serology. All examined serum samples (n = 25) revealed positive results in PCR, while only ten samples were positive in serology (Figure 1). All serum samples collected from aborted cows (n = 10), buffaloes (n = 5), ewe (n = 1) and goats (n = 9) were positive with the genus specific bcsp31 real-time PCR assays. Interestingly, *B. abortus* DNA was identified in all serum samples collected from cows, buffaloes, ewe and goats. It is worth mentioning that one ovine serum contained both, *B. abortus* and *B. melitensis* DNA (Table 2). Bacterial isolation failed to isolate *Brucella.*

**Table 2:** Serology and real-time PCR results of serum samples collected from animals, which had aborted recently and positive in at least one test

Case no.	Host	History of samples collection after abortion (weeks)	Serological assay			PCR^d^		
			RBT^a^	CFT^b^	ELISA^c^	Bcsp 31	IS711 *B. abortus*	IS711 *B. melitensis*
1	Cow	4	+	+	+	+	+	−
2	Cow	3	+	+	+	+	+	−
3	Cow	6	+	+	+	+	+	−
4	Cow	4	+	+	+	+	+	−
5	Cow	3	+	+	+	+	+	−
6	Cow	3	+	+	+	+	+	−
7	Cow	6	+	+	+	+	+	−
8	Cow	4	+	+	+	+	+	−
9	Cow	4	+	+	+	+	+	−
10	Cow	3	+	+	+	+	+	−
11	Buffalo	4	+	+	+	+	+	−
12	Buffalo	1	−	−	−	+	+	−
13	Buffalo	1	−	−	−	+	+	−
14	Buffalo	1	−	−	−	+	+	−
15	Buffalo	1	−	−	−	+	+	−
16	Goat	4	+	+	+	+	+	−
17	Goat	3	+	+	+	+	+	−
18	Goat	4	+	+	+	+	+	−
19	Goat	1	−	−	−	+	+	−
20	Goat	1	−	−	−	+	+	−
21	Goat	1	−	−	−	+	+	−
22	Goat	1	−	−	−	+	+	−
23	Goat	1	−	−	−	+	+	−
24	Goat	1	−	−	−	+	+	−
25	Sheep	4	+	+	+	+	+	+
Total positive			15	15	15	25	25	1

^a^Considered positive when showing any degree of agglutination.

^b^Positive samples (≥20 IU/ml).

^c^positive samples showing cut off values (≥2).

^d^Positive samples showing ct value (ct ≤ 40).

## Discussion

In developing countries such as Egypt, conventional tests done on serum are used for screening of brucellosis and play an important role in surveillance programs of the disease [13]. Based on previous publication about brucellosis in Egypt, this study is the first to record *B. abortus* DNA in sera samples of sheep and goat. *Brucella* organisms were not isolated in this study. *Brucella* culturing is hazardous, and the technique is restricted to few laboratories in Egypt. Isolation rate is very low even in experienced laboratories [13]. The probability of successful isolation of *B. abortus* is markedly reduced when a few organisms are present in the samples or the material is heavily contaminated. Negative culture results cannot exclude infection with *Brucella* [15]. Nevertheless, clinical presentation i.e. abortion and strong seropositive results finally led to the diagnosis of brucellosis. Serological diagnosis from freshly aborted animals may fail because antibody titers against *B. abortus* rise only 1–2 weeks after infection [16], however circulating *Brucella* DNA may be detected with molecular techniques. These facts can explain the absences of antibody titres in some animals. Serological diagnosis of brucellosis is presumptive evidence of infection and laboratory confirmation of brucellosis requires isolation of bacteria or detection of *Brucella* DNA by PCR. Thus, the diagnostic window of *Brucella* serology should be complemented by bacteriological or molecular diagnosis [17]. PCR assay able to detect *Brucella* DNA in seronegative animals and it was proposed to use PCR even as a tool for routine diagnosis [18]. Our results corroborate this proposal.

All *Brucella* species are closely related and can be considered as pathovars of a single species [19]. Thus, it is not unexpected that host specificity of *Brucella* spp. is not ‘absolute’ but ‘relative’ [1]. Although ruminants in general are susceptible to *B. abortus,* the infection in small ruminants is rare [1]. Experimental infection of pregnant ewes with *B. abortus* produced late term abortions. The aborted ovine fetuses developed lesions due to systemic infections similar to those reported in bovine fetuses after natural and experimental infections [20]. *B. abortus* infections have been reported in sheep in the USA [21], in Nigeria [22, 23] and in Iran [24]. The protective efficacy of vaccines against *B. abortus* infections has not been studied in small ruminants and may play a role for the persistence of brucellosis in cattle [1, 25, 26]. In Egypt, *B. abortus* bv one and three have been reported in cattle and buffaloes [12, 27]. Cross species transmission of *B. melitensis* to cattle and buffalo from small ruminants that shared the same stables and farmyards was recognized in Egypt [10, 28, 29]. Recently, *B. melitensis* DNA was also detected in milk samples collected from apparently healthy cattle and buffaloes by real-time PCR [30]. However, no reports could be found that *B. abortus* or its DNA was ever found in small ruminants in Egypt. To the best of our knowledge; this is the first report of sheep and goat brucellosis caused by *B. abortus* in Egypt. Accidental *B. abortus* infections in small ruminants may even play an understanding role for the persistence of brucellosis in cattle [1].

Detection of both, *B.abortus* and *B.melitensis* DNA, in one animal observed in this study demonstrated that one host can be infected with two different species of *Brucella* at the same time. The potential host range of Brucellae may also depend on breeding conditions [19]. Co-habitation and close contact of different animal species increase the risk of a pathogen to cross the species barrier [31]. Infection of small ruminants with *B. abortus* can occur as result of natural exposure to infected materials from another species or indirectly through contact with soil contaminated with abortion secrets. Brucellae can survive up to 15–25 days on a pasture depending on environmental conditions e.g. intensity of UV-light [31]. It is likely that the Egyptian Baladi goats and sheep which had aborted had contact with either the fetus or infective fluids from cattle abortion. Isolation of *B. abortus* DNA from a doe that aborted corroborates a cross-species transmission of the *Brucella* spp.

## Conclusion

In summary, clinical presentation i.e. abortion and presence of *Brucella* DNA finally led to the diagnosis of brucellosis caused by *B. abortus* in Egyptian Baladi does *(Capra hircus)* and sheep (*Ovis orientalis aries*). To the best of our knowledge, our study is the first record on brucellosis caused by *B. abortus* in small ruminants in Egypt. Our findings indicate also that, in endemic areas like Egypt, where both *Brucella* spp. are present and small ruminants are raised with cattle in close contact in the same pasture, transmission of host specific *Brucella* species to non-preferred hosts may occur. These results should be taken in account while assessing the epidemiological situation in an area and during implementation of control measures. Trials to isolate the bacteria and molecular typing such as multi-locus variable number of tandem repeats (MLVA) to obtain an epidemiological evidence of transmission between animals is required.

## Abbreviations

RT-PCR: real-time polymerase chain reaction
RBT: rose bengal test
CFT: complement fixation test
ELISA: enzyme linked immunosorbent assay
MLVA: multi-locus variable number of tandem repeats analysis
